# Exploring football coaches’ views on *coach education*, *role*, and *practice design*: An Australian perspective

**DOI:** 10.1371/journal.pone.0285871

**Published:** 2023-05-16

**Authors:** Erch Selimi, Alexandra Lascu, Fabio Serpiello, Carl T. Woods

**Affiliations:** 1 Institute for Health and Sport, Victoria University, Melbourne, Australia; 2 Research Institute of Sport and Exercise, The University of Canberra, Canberra Australia; 3 Department of Sport and Exercise Science, James Cook University, Townsville, Australia; Mugla Sitki Kocman University: Mugla Sitki Kocman Universitesi, TURKEY

## Abstract

Despite the importance placed on the design and delivery of formal coach education programs by Football Australia, there remains a lack of research relating to how formal coach education strategies support Australian football (i.e., soccer) coaches and their coaching practices. Through a series of semi-structured interviews, 20 highly qualified and experienced Australian senior football coaches shared their perspectives on: (i) *coach education*, (ii) their *role as coach*, and (iii) *practice design*. Findings revealed that formal coach education in Australia was largely ineffective in preparing senior coaches for the realities of senior football. Coaches attributed this to a number of factors, including the content’s quality, structure and delivery, which they viewed as rudimentary, outdated, repetitive and lacking in relevance and depth. Coaches also revealed there was an expectation of conformity to the content and practices endorsed by the National Football Curriculum, limiting the value and impact of formal coach education in supporting the development of coaches’ theoretical and practical dispositions. These findings point towards a number of broad and systemic issues relating to the conceptual, theoretical and practical foundations of the National Football Curriculum and subsequent courses. If Football Australia are to reach their goal in designing and delivering effective and meaningful coach education programs that support the highly complex and multifaceted role of senior coaching, formal coach education may need to adapt and evolve in a manner that better supports the multi-dimensional and context-specific needs of Australian senior football coaches.

## Introduction

Sports coaching is a complex task [1]. Socio-cultural constraints operating at relatively long timescales (i.e., sporting identity, club culture, path dependent behaviour within an organisation) are likely to implicate how a coach views their role when situated within a broader ecology of relations [2,3]. While at shorter timescales, the pressure to win and perform (especially in high-performance sport), coupled with consideration of other stakeholders (i.e., players and support staff), likely implicates how coaches go about taking up with the day-to-day nuances of their role [2,3]. Football (i.e., soccer) coaching is no different. Coaches are required to operate in complex socio-cultural environments, while attempting to implement effective performance preparation frameworks at daily, weekly, monthly, and even yearly scales. Such complexity, in part, has contributed to the recent growth of interest–both practically and academically–in the professional education and development of sport coaches [4,5].

In 2020, Football Australia (FA)–the national governing body overseeing the development of football in Australia–released the ‘*XI Principles for the future of Australian Football*’. This document outlines eleven fundamental principles for the future growth and development of football in Australia [6]. Of particular interest to this study is *‘principle six’*, titled: *‘Create world class environments for coach development’*. In an effort to raise the standard of coaching and coach development, a number of promising measures have been proposed by FA, including: the need to foster a strong culture of coach development by emphasising the role’s importance in player development, modernise the method of coach education delivery, and review the content of coach education courses and the Australian coaching methodology [6]. At present, the Australian football coach education system is underpinned by the *National Football Curriculum* (NFC). Released in 2009, and updated in 2013, the NFC aims to provide coaches with an understanding of the national ‘playing’ and ‘coaching’ philosophy, advocating for a i) player-centred approach to coaching; ii) game-based, constraints-led approach to practice design; and iii) an information-processing view of motor learning [7]. Described by FA as an ‘holistic’ approach to coaching, FA hoped to establish a coaching culture where the implementation of theoretically-informed and evidence-based practice was a central component of coaching [7]. Despite the importance placed on formal education and accreditation by FA, there remains a relative paucity of research addressing how FA’s formal coach education strategies and the NFC support coaches and their coaching practices.

Currently, the football coach education system in Australia offers two distinct development pathways: the Community and Advanced pathways. The Community Coaching pathway is tailored toward coaches of participation players, aged between 5 to 17 years. Courses are relatively short, running for 1 to 2 days, with the content primarily focused on providing coaches with practice design and delivery guidelines [7]. According to studies that have observed coaching practices in such settings, although practice sessions are largely structured in accordance with NFC recommendations, coaching practices and behaviours are generally not reflective of the player-centred, constraints-led approach promoted by FA [8,9]. While findings from these studies have been important, such insights have mostly been based on community coaches with limited experience and accreditation. As such, the findings may not offer a representative view of the quality of coach education and content within the NFC.

The Advanced Coaching pathway is primarily targeted toward those working in the performance phase of football. Courses are more intensive than the Community pathway, often requiring 10 to 14 contact days, with content designed to cover FA’s Coaching Expertise Model that outlines a list of themed competencies (*training*, *the match*, and *management*) that a coach must acquire [10]. There are four levels of senior accreditation offered: the ‘C’, ‘B’, and ‘A’ licence, and a ‘Pro Diploma’, with the central tenet of this process aimed at ensuring the NFC’s coaching principles are easily understood and brought to life [10]. As such, the ‘C’ and ‘B’ licence are designed in a manner that encourages coaches to adopt FA’s vision and philosophy. Only at the ‘A’ licence level are coaches encouraged to develop and establish their own vision and philosophy of the game, which upon completing, are expected to be able to clearly articulate [10]. In addition, coaches aiming to work at the National Premier League level or higher are required to hold at least a ‘C’ licence, further signifying the important role FA’s formal coach education system has on Australian coach’s development and accreditation journey [7].

Globally, formal coach accreditation processes are thought to be important for quality assurance, ensuring a baseline of knowledge and competency across a variety of coaching cohorts [11–14]. As such, numerous countries have developed national coaching curricula [15], aiming to assist coaches “hone in their craft and become effective and ethical at achieving desirable ideals” [4 p.2]. This increase in the provision of formal coach education is due, in part, to the professionalisation of modern-day coaching, where coaches are now tasked with greater responsibilities, both on and off the pitch [16]. For example, professional coaches are often overseeing, not just the on-field performance of players, but are likely having a significant input to their off-field development, manifest through well-being and player welfare. As such, there is a growing consensus within high-performance sport that coaches are not just individuals who (co)design game plans and devise training strategies, but are *managers of people*–both players and support staff [17–19]. These emerging requirements of professional coaches point toward one of the many reasons we have seen an increase in the level of interest in the development of coach education programs from a variety of stakeholders [20], leading to a more scrutinised and regulated industry [21].

Given this, formal coach education, for all intents and purposes, is considered an integral and necessary component of a coaches developmental journey [22,23]. Despite significant empirical reports, the transfer of theoretical knowledge from formal coach education to the pitch has been difficult to demonstrate [4]. This could be related to the lack of alignment coaches feel with the theoretical material being covered [11]. Further, a continued and over-balanced focus on technical and strategical aspects of the game could limit the development of pedagogical and interpersonal competencies coaches feel are required to ‘do’ their job effectively [13,24–27]. The complex nature of coaching not only makes formal coach education challenging to implement [28], but “seriously calls into question the legitimacy and value of an overly-instrumental approach to coach learning and its provision” [11 p.3]. Critiquing along similar lines, Nelson [29] referred to formal education settings as a process more aligned to ‘indoctrination’ rather than ‘education’, while Abraham [30] suggested that formal coach education is often caught attempting to convince coaches of a singular and appropriate way of coaching, preventing exploration. This risks a view of formal coach development and accreditation regressing into a ‘box ticking’ exercise, rather than a valuable and integral part of their development journey. Formal coach education–*in its current state*–may thus not be able to foster the development of all the key skills the multi-faceted role requires [24,31–33]. To this end, the aim of the current study was to explore: (i) how Australian football coaches take up with formal coach education, (ii) how Australian football coaches conceptualise their role with regards to performance preparation; and (iii) how/why Australian football coaches use various practice strategies to support player development.

## Method

### Study design

This study used an interpretivist, qualitative research design. This interpretivist position allowed for coaches to articulate unique meanings of their experiences and practices within their coaching domain [34], appreciating that the understanding of individual perspectives can only occur through observation and interpretation [35]. Ethical considerations for the research project in line with the National Statement on Ethical Conduct in Human Research was undertaken [36]. Additionally, a detailed ethics proposal was submitted and approved by the Victoria University Human Research Ethics Committee (Reference number: HRE22-031).

### Participants

A purposive, criterion-based sample of Australian football coaches (n = 20) were recruited to participate in this study. In order to provide a comprehensive and well-founded account of their experiences during FA’s coaching courses and subsequent coaching content, coaches were required to have, at a minimum: i) an FA ‘A’ licence, and ii) accumulated 3 years of senior coaching experience (see [19]) within Australia. Altogether, 16 coaches held an ‘A’ licence, and 4 held a ‘Pro Diploma’. The sample level of senior coaching experience at the time of the interviews (defined temporally in years), ranged from 4 to 30 years.

Participant recruitment involved utilising existing networks within the Australian football community, social media posts, messages, emails, or through face-to-face conversation. After initial contact, participants received information regarding the project, example topics of conversation, and were able to ask questions to clarify the interview or data analysis process prior to an interview being organised. Written consent was obtained from participants prior to the interview starting. Consent was provided on the understanding that interviews would be anonymised. Thus, participant identities were coded numerically throughout all analysis (defined from HC1–HC20).

### Data collection

Data was collected via semi-structured interviews that focused on three pre-defined themes related to the studies aims: *coach education*, their *role as coach*, and *practice design*. The questions were pre-planned yet open-ended, which allowed the conversation to flow naturally in a direction guided by the key themes [37]. The open-ended nature of the questions also provided this study with a broader understanding of the topics discussed while enabling participants to provide richer and deeper insight relative to close-ended interviews [38]. The questions used to guide the flow of the semi-structured interview are detailed in Table 1.

**Table 1:** The themes and subsequent questions explored in the semi-structured interviews.

Theme	Questions
Coach Education	How would you describe your experiences on the coaching courses?
	What were your thoughts on the content delivered on the courses?
	What did you learn from the coaching courses?
	Do you feel the content delivered, prepared you for senior coaching?
Coaches’ Role	What is coaching to you?
	How would you describe your coaching approach?
	What do you consider your main role is as senior coach?
	What skills do you need to be an effective coach at senior level?
Practice Design	How do you approach practice design?
	What type of training do you feel best prepares players for the game?
	What do you think is the most effective way to develop skilful players?
	How do you view your role with regards to performance preparation?

The first author reflected on the interview process after each interview in order to evaluate the structure and appropriateness of questions. This resulted in only very minor modifications to basic wording, with no other changes made following reflection. All interviews were conducted by the first author online or in person between May and September, 2022. The duration of the interviews ranged from 73 to 145 minutes and were recorded for subsequent transcription and analysis.

### Data analysis

Interviews were transcribed verbatim in Trint (Trint Ltd) by the first author. A pragmatic, two-staged thematic analysis was then used to analyse the data [39]. The first coding stage included an inductive approach, which entailed data immersion by re-reading transcripts and re-listening to interview recordings, as well as highlighting any interesting quotes or passages. Highlighted content was exported to Excel, where each excerpt was assigned a key code or description that encapsulated the ’essence’ of the material. The second author then analysed and examined the emerging codes and their relationships for consistency and relevance. This process of researcher-triangulation enabled the first author’s original codes and interpretations to be challenged and probed, ensuring that (i) codes could be appropriately organised into themes and (ii) the personal experiences and biases from the primary author did not lead to potential misinterpretations and distortions of the data [40]. Given that the primary author had previously participated in FA’s Advanced Coaching pathway and was a practicing coach, this reflexivity was essential for establishing and reinforcing the data’s trustworthiness and authenticity [41]. Next, a deductive analysis was conducted to compare the literature on (i) *formal coach education*; (ii) *coaches’ roles*; (iii) *practice design*, as well as the emergent themes discussed by the participants. All categories and emergent themes are presented in the results and discussion section, accompanied by selected quotes from the interviews. Thus, for brevity, both the results and discussion are presented concurrently.

### Results and discussion

Given the magnitude of data collected, only excerpts that the authors felt best captured and reflected the sentiments expressed by the participants were included. The presentation of results and discussion section are guided by the three pre-determined themes: (i) perspectives on formal coach education, (ii) coaches’ role, and (iii) practice design. Each theme is composed of sub-themes that are either: (i) representative of the specific pre-determined topics discussed or, (ii) a collection of recurring themes that emerged from the openness of the interviews.

### Theme 1: Perspectives on coach education

The first theme to be explored relates to coaches’ perspectives on formal coach education in Australian football. According to FA, there are three major areas of competency that senior coaches must develop: *training*, *the match*, and *management* [10]. As such, the interview questions were developed around these three sub-themes of coach education, while concurrently affording opportunities for participants to elaborate on their general coach education experiences as they felt necessary.

#### Sub-theme 1.1: Preparing coaches for senior football

National sporting organisations, such as FA, oft-attempt to support the development of coaches by providing well-designed and applicable coach education programmes [1]. Hence, there was a particular interest in the role formal coach education played in preparing coaches for the realities of senior football. A common sentiment shared among participants was the influence of the courses, particularly the ‘C’ and ‘B’ licences, in providing them with a framework to help plan and structure training sessions effectively. This is exemplified by an excerpt from HC18, who stated:

*“I was pretty green and raw going into the whole coach education process*. *I felt like I really needed something that I could hang my hat on and use as a starting point to then progress forward from. I had some ideas based on my coaching, but I wasn’t sure how to translate those necessarily across onto a football field..…it just provided me with the structure that I needed, it gave me a framework and a tool kit that I could lean on.”*

This sentiment is in keeping with the literature (see [8]), and should come as little surprise, given that FA’s earliest Advanced Coaching Courses are largely guided by *’The Football Coaching Process’*, which is a document designed to support coaches in planning, structuring, and delivering practice sessions [10]. Given there is suggestion that coaches practice their craft with little reference to a coaching process [42], FA seem to have good reason to focus on delivering a framework that helps coaches’ plan and structure training sessions. Though, despite participants expressing positive sentiments about the formalities of coach education on their basic practices, particularly in planning and structuring their training sessions, the changes in their coaching makeup seemed to be more *methodological* than *theoretical*. This was emphasised by HC14:

*“When you’re starting out*, *it gives you all the tools that you need to make sure that the perception is that you know what you’re doing, you’re organised, and it seems like you’re doing a good job.”*

In other words, the positive sentiments expressed by the participants seemed to point towards basic procedural developments in their practices. This was demonstrated in the fact that participants often remarked on the ineffectiveness their formal education had on preparing them for the role of senior coaching. This reinforces the notion that the senior coaches role extends beyond the development, design, and implementation of game plans and training sessions [43]. It also requires strong inter- and intra-personal competencies in order to cater for the diverse social contexts they find themselves in [18]. A sentiment also shared by HC13:

***“**I don’t think the A licence prepared you [for senior coaching] to be honest. It was meant to be preparation for a professional coaching career, the qualification required to coach in the A-League as an assistant*. *But I don’t think it prepares you for that. If you’ve never been in that environment, there’s still a lot of learning to do when you get in there. So, I think it does leave you underprepared from a management perspective and what to expect around that environment. But it does give you preparation in terms of the daily process of training and giving you clarity in terms of a football process and identifying what you want to work on.”*

These views are in keeping with reports that formal education has typically left coaches feeling unprepared for the rigours of senior coaching (see [44,45]). This is expected, though, given that coaching courses have traditionally been criticised for delivering abstract content far removed from current practice [11]. If the objective of formal coach education is to develop coaching knowledge, behaviours, and practices, then it seems important for FA to find a balance between the ‘development of coaches’ and the ‘accreditation of coaches’. As noted in the excerpts presented, these two things are not the same.

Guiding the development of coaches is FA’s Coaching Expertise Model, found within the NFC, which outlines a list of competencies (*training*, *the match*, and *management*) and football knowledge (*principles of play*, *strategy*, and *tactics*) that are considered essential to become an ‘expert coach’ [7]. Though, despite the importance placed by FA on the attainment of football knowledge within this model, participants felt the courses did not adequately address this area and were somewhat dissatisfied with its delivery:

*“They [Coach Educators] always assumed that you had the football knowledge when you first come in. That was sort of the prerequisite*. *They thought you should just know. So, I think there could be a lot more work done in terms of developing football knowledge.” [HC11]*

*“I think that [the lack of football knowledge] is probably the biggest weakness in terms of content across all of the courses.” [HC15]*

When asked about why the development and attainment of football knowledge was an important feature of senior coaching, most participants were of the view that a deep knowledge *about* the game (see [46]) was essential for supporting the growth of a player’s knowledge *of* the game [46]. As HC12 explained:

*“The reason [knowledge about football is important] is they’ll [players] call your bluff in two seconds. So, if I was to have no football knowledge*, *in the difficult times when you’re down at half time, when the players need something from you that gives them credible evidence, a tangible thing to take away that can improve their own and their team’s performance, and if you don’t provide it, you’ll get called out in two seconds. I think it’s [knowledge about football] absolutely key to the coaching toolkit. Football people are football people and if you’re not a football person, unless you’re Ted Lasso, it’s not happening for you.”*

The importance placed on the acquisition of football knowledge is supported in the literature [18]. Moreover, the appreciation of football knowledge is conveyed by FA, who suggest “without in-depth football knowledge, the quality of what the coach does will be adversely affected” [10 p.13]. Despite this, ‘football knowledge’ is not considered as one of the three pillars of competency. Instead, FA encourage that ‘football knowledge’ be acquired through a variety of unmediated and informal ways such as: ‘*playing’*, *‘coaching’*, *‘analysing*’, and *‘talking’* about football [10]. However, although, coaches express a strong appreciation between football knowledge and coaching expertise (see [19]), research suggests that the attainment of football knowledge alone is inadequate to become an effective coach [17,47]. Rather, the development of football knowledge coupled with interpersonal and intrapersonal skills are regarded as an essential requirement for an effective coach [18]. Referred to as ‘*management skills*’ by many of the participants interviewed, this was another area participants believed the courses did not adequately address. For example, HC8 and HC14 stated respectively:

*“The courses have helped with the coaching process, but the coaching process is only one aspect. How you deal with people*, *how you respond to setbacks and challenges is another thing and that’s a big part of coaching. And that’s not catered to enough in the courses.”*

*“I almost feel like it’s back to front in the management area and learning how to relate and be in a group of people*. *Those types of [management] skills….I feel like there’s a whole piece missing there or it’s too late in its introduction.”*

These findings are in keeping with the literature, which indicates that the support of inter-personal skill is disproportionately supported relative to sport-specific knowledge [48]. Although FA views ’management’ as a key pillar of coaching competency, and its delivery is heavily prioritised in the ‘Pro Diploma’, participants voiced feelings of dissatisfaction with the amount of attention and content devoted to management. Consequently, it may be beneficial for FA to reconsider the placement, delivery and the amount of time spent on developing management skills during the courses. Crucially, these findings point towards a potential disconnect between FA and the participants’ perceptions of the competencies required to be considered an *effective* senior coach and, perhaps more importantly, how coaching competency should be *assessed*. As HC7 discussed:

*“Had a coach educator come to where I was working and spent a week with me, for example, in the planning process and in training sessions and team meetings on game day, you get a much better window into who the coach is*. *Are they an A licenced coach?, maybe or maybe not. But you can tell very quickly if that person is going to be a good coach or not. And that should be the basis of passing or failing or recommending further development. But at the moment, you’re in a group of 30 to 40 people, maybe you contribute once or twice in the classroom, you put on a session nervously, with a load of guys that don’t really want to be there. It either goes well, or if it doesn’t, you fail and then you’re suddenly not in A licence coach. Or, you have a personal relationship with the coach educator, or you’re an ex-socceroo and suddenly you’re an A licenced coach. Yeah, it’s pretty backwards.”*

This sentiment highlights the challenge FA have in *developing* and *assessing* coach competency in its current format. Indeed, participants often felt like they were “*acting*” [HC19], eluding that the courses were merely a “*box ticking exercise*” [HC16] that had minimal influence on their coaching approach. Such findings are in agreement with the literature, in that coaches undertaking formal coach education are often seen engaging in ’studentship’ tactics to convey qualities desired to pass the course [44]. To address this issue, participants in our study remarked on how forms of *mentorship* within the coach education process “*could add another layer of value to the coach education process”* [HC15], providing a more *“learner-friendly environment that allows you to be a lot more expressive*, *leading to a more personalised assessment of competency*” [HC7]. Indeed, it has been suggested that coaching competency can be better *understood* and *developed* in a coaches respective coaching environment [49], implying that the *assessment* of coaching competency may also need to take place in such representative settings. Thus, in order for formal coach education to meet the needs of senior coaches, FA may need to re-conceptualise and re-structure the *development* and *assessment* of coaching competency, permitting learner-centred approaches (i.e., mentor- apprenticeship) to guide coach development.

#### Sub-theme 1.2: Coaches’ perceptions of the course content

According to the majority of participants, the quality of content presented on the courses was one of the key reasons why they felt formal coach education did not prepare them effectively for senior coaching. The content on the ‘C’, ‘B’, and ‘A’ licences was described as “*rudimentary*” [HC8, HC16], “*outdated*” [HC20], “*repetitive*” [HC3, HC4, HC5, HC13, HC18, HC19], and needing “*an absolute revamp*” [HC12]. This is further reiterated by HC7:

*“I’m certainly not afraid to say that the biggest jump I made as a coach was doing the C licence, and from there I kind of thought, I’ll go into the B, A, and the Pro, and I’m going to make all these huge leaps*. *But the reality is the B was really an extension of the C, and the A an extension of the B…I was underwhelmed by the B and the A. I think the learning was very minimal, the content, structure and methodology of coach education was disappointing. But as we know, it’s a box that everybody has to tick. And it certainly felt like everybody was there just to see the time out and get their boxes ticked.”*

The participants perspective that the course content lacked relevance was one of the most frequently cited reasons for their dissatisfaction. Participants revealed that they performed under vastly different contexts and conditions than those presented by the NFC, making the content challenging to connect and identify with. The following remarks capture this position:

*“A coach looks at it [the NFC] and says, I’m under pressure, we’re in the bottom half of the table, we’re closer to relegation than we are to winning the competition, and people are telling me I should have this philosophy because that’s what the national curriculum says…? That’s nonsense, it erodes the confidence of people*. *These procedural documents that define what the game should be…it doesn’t match the reality. I think that disenfranchises people that are saying, well, in those circumstances it doesn’t apply to me, it’s irrelevant to me. I just need to survive.” [HC3]*

These sentiments are consistent with the literature, in which the decontextualised manner of formal coach education means that the unique context that each coach operates in is usually ignored and disregarded, resulting in a lack of relevance and significance for coaches [31,50]. This points to one of the biggest challenges facing the make-up and delivery of content within formal coach education; if it is unable to meet the individual needs of coaches, it is likely that many will simply ignore the content delivered and continue to practice as they have in the past [51]. Thus, FA may need to reconceptualise the content within the NFC to better reflect the daily needs of senior coaching.

Beyond the participants dissatisfaction with the quality and relevance of the content delivered, there was an overwhelming sentiment that perhaps the most frustrating feature of the coach education process was the lack of freedom to explore. The expectation to *conform* was denoted amongst most participants, even into the later stages of the ‘A’ licence. It is, thus, worth quoting three participants at length here, given the richness of their responses related to feelings of constraint:

*“If you take that freedom away from people when they’re going through the coach education process*, *the freedom to think differently, you’re kind of just indoctrinating in a certain way of thinking, and I’m not sure that’s what we should be trying to do.” [HC3]*

*“I felt like it was very repetitious. It felt like everybody was trying to do the same thing on the courses*. *It was very homogeneous. It was almost expected that you had to toe the line and coach a certain way in order to pass the course. And again, I just don’t know if that’s conducive to robust learning and becoming a better coaching community if we’re going to do it that way*. *I just felt we could have had more different types of sessions or contrasted more with the national curriculum to sort of make you appreciate it or understand its weaknesses more in coaching that way.” [HC16]*

*“I think that we have tried to take that [exploration] away from a lot of coaches and make them all cookie cutter coaches*. *And then you get robots and nasty, sterile coaches that, you know, if everybody’s learning to coach the same way, how are they going to adapt when they come up against something different? I think that’s one thing that was missing from the courses, right the way through.” [HC4]*

These responses are unsurprising given that the ‘C’ and ‘B’ licences are explicitly designed to encourage coaches to adopt FA’s vision and philosophy. There is an expectation from FA that by the time coaches progress to the ‘A’ Licence or ‘Pro Diploma’, they should be able to develop and articulate their own philosophy [10]. Despite this stance, participants believed that the ’A’ and ’Pro’ licences were still heavily based on the NFC, limiting their ability to explore, develop, and expand their vision and philosophy of the game. These sentiments are prevalent in the literature, where coach education is frequently accused of *indoctrination*, merely employing a one-size-fits-all approach [50]. Accordingly, if FA are to realise their goal of developing ‘world-class’ environments for coach development [6], then formal coach education may have to move beyond a standardised football curricula based on transmission and application–giving coaches a ‘toolbox’ of professional knowledge–and aim toward a deeper, more meaningful change that encourages search and exploration.

#### Sub-theme 1.3: Coaches’ perceptions on the delivery of content

Recent literature has emphasised the important role coach developers have in the delivery of formal coach education content [52–54]. However, for the participants in this study, coach developers were seen as “*good guys and professional coaches*, *but not always great educators… you can tell the [coach developer] never taught anybody anything*” [HC12]. Coach developers were considered generally ineffective at communicating, presenting, and delivering content, as further discussed by HC7:

*“The message between the curriculum and the application in the coach education space was really lacking*. *I saw coach educators who were so authoritative and rigid and structured and upon reflection, I thought, now this wasn’t meant to be applied like this. And somewhere the message has been lost.”*

Although the above reflections are noteworthy, some assert that much of the criticism levelled at coach developers can be attributed to broader, systemic issues that reside beyond their control (i.e., situating ‘education’ in a model of transmission, not primary experience and careful exposure) [55]. While we are cautious about drawing too negative a conclusion about the (in)effectiveness of coach developers here, our findings do suggest that future work needs to examine ways in which coach developers working within FA’s coach education pathways can be better supported in developing, structuring, and presenting course content.

### Theme 2: Conceptualisation of the coaching role

The next theme explored the conceptualisation of the coach’s role, which will concurrently encapsulate coaching approach, expertise, and knowledge needed for senior coaching. This component of the interview allowed participants to share their beliefs toward coaching and its function within a senior football environment.

#### Sub-theme 2.1: Coaching is multifaceted

A football coach’s roles and responsibilities are vast in both scale and scope [56]. Because of the multifaceted nature of coaching, coaches are frequently required to develop knowledge and expertise in a variety of fields, which can often be found outside their sport [33]. This was no different for the participants in this study, as noted by HC15:

*“It’s a mixture of stuff. It’s a leader. It’s a motivator. It’s a CEO. It’s a mentor*. *It’s a father. It’s a brother. It’s not one thing. It’s a moving target. And you need to be robust in your makeup as a person, psychologically and emotionally and in every way. You need to be sensitive to the environment around you. You can’t be oblivious to it. It’s a whole lot of stuff. And then by the way, you need a little bit of football knowledge because, that at the end is the bit we’re doing.”*

This response highlights the multi-dimensional nature of ‘coaching’ at the high-performance level. For the vast majority of participants, articulating the role of the senior coach was difficult, resulting in lengthy responses that further illustrated the role’s complexity. This could also be said of FA, whose description of the senior coach’s role–“to prepare successful teams” [10 p.58]–may considerably understate the role’s scope. Perhaps it is apt to question whether the title ‘coach’ is adequate in describing the wide range of duties and responsibilities that an individual may undertake and be accountable for, particularly in performance phase settings? Nash [43] supported this notion, arguing that due to the role’s uniqueness and idiosyncratic features, no definitive description can be established. Regardless, gaining deeper insight into the complexity of coaching, as well as the varying needs associated with different contexts is critical if FA are to design coach education programs reflective of the scenarios and contexts senior coaches find themselves in.

#### Sub-theme 2.2: Coaching approach

As stated earlier, FA advocate for a ‘player-centred approach’ to coaching [7]. Defined by “a style of coaching that promotes athlete learning through athlete ownership, responsibility, initiative and awareness, guided by the coach” [57 p.1], player-centric coaching practices actively encourage players to be involved in the decision-making and problem-solving process. While participants generally favoured player-centrism, the nature of high-performance football and subsequent pressure appeared to make it challenging for them to consistently take up with such an approach. Some participants were “*happy to have a little bit of dialogue with players*, *but when push comes to shove*, *and I say it’s going to be that way*, *it’s got to be that way”* [HC11]. An excerpt from HC8 discusses this in depth:

*“I try to be very inclusive and be a facilitator. I think on reflection, if I coach in a senior environment again, [I’ll need to be] more strict, more dominating, for lack of a better word*. *And when I say more, I only mean a little bit, I don’t mean I should go to the complete opposite end of the scale, but I feel like I was too nice or too flexible, allowing them to take the lead. Although there’s massive benefits to having that (player-centred) approach and engaging your playing group, you still have to be the ultimate authority. You have to have someone who can make a final call, who has the respect from people under them to make that call, and then they will follow that person’s decision.”*

Although participants saw the advantages of supporting greater player ownership and accountability through a player-centred approach, the apparent socio-cultural constraints of high-performance football seemed to make this difficult to implement on a consistent basis. These sentiments are consistent with the literature, which suggest that coaches who work in high-pressure contexts are more likely to regress to controlling, directive, and prescriptive coaching [57–59]. If FA are to progress with a player-centric approach, then it is vital that they more clearly show how this can be achieved within the socio-cultural constraints of high-performance football.

#### Sub-theme 2.3: Management and interpersonal skills

Senior football coaches are required to interact frequently with a broad range of stakeholders. Accordingly, it has been suggested that a coaches’ effectiveness is largely dependent on their capacity to nurture meaningful and productive relationships [18]. This sentiment was also shared by participants here. Namely, despite the role requiring a diverse set of competencies, the ability to help develop people and build sustainable relationships appeared key to the senior role:

*“Coaching is all about relationships. There are other facets to it, like the way that you communicate, deliver a message and the process you go through but I think without strong relationships you are doomed to fail because no matter how knowledgeable you are at your craft*, *if you can’t connect with someone, the chances of them hearing your message, let alone taking it on board and running with it, are very limited" [HC18]*

**“***The role is to develop people. You have to be a better person after you’ve come through that environment. They have got to be a better footballer, a better personality, a better leader*. *And then under all of that is winning of course, you’ve got to win. Because, if you’re a senior football coach, you’re there to win games or you’re there to win relative to whatever your objectives are. So, that’s a non-negotiable part of being a senior coach.” [HC11]*

This appears to align with the literature, which suggests that coaches who invest time in building strong relationships with players are likely to promote a connected and interactive environment that supports player development [1,13,60]. Moreover, considering all participants had coached teams that were focused heavily on performance outcomes (i.e., winning), they each seemed appreciative that team cohesion and performance outcome were not mutually exclusive. This is of note, although FA consider ‘management’ to be one of the key pillars a coach needs to be competent in, such skills are only briefly touched on in ‘*The Football Coaching Process*’ [10], and may not be sufficient in supporting coaches at the required level. Our results suggest that further emphasis and investment in supporting the development of management skills (i.e., interpersonal, and intrapersonal) could be of use within the coach accreditation process in Australian football.

#### Sub-theme 2.4: Delivering a vision, playing style, and getting results

Senior coaches in high-performance sport are largely evaluated on the results obtained. In other words, coaches are expected to “*win football matches*, *first and foremost*” [HC4]. Therefore, it should not be surprising that the need to produce results is a significant source of pressure and focal point for senior coaches [59]. Our findings showed that given this pressure, the importance of the curriculum diminishes overtime: “*senior coaches don’t care about the curriculum*, *they care about results*” [HC8]. This sentiment was also shared by HC2:

*“You need to get the results first before looking to develop players*. *Unless you’ve come from a previous background where you’ve had some good results or you’ve been a high-profile player, I think you get a little bit more time then. But if you’re a young coach and no one knows you, you have to pretty much get results straight away.”*

Despite the importance of getting results, there is a growing expectation within the football community that coaches also need to deliver a ‘club vision’ and ‘playing style’. This belief is echoed by FA, who have summarised Australia’s preferred playing style in the following statement: *“A pro-active brand of football*, *based on effective possession*, *with the cutting edge provided by creative individuals*. *Defensively the key components are quick transition and intelligent collective pressing”* [7 p.17]. Participants expressed an expectation and pressure “*to be competent enough to articulate your vision and your playing style*” [HC11] such that supporters, the club, and other stakeholders could identify with it. Thus, it appears important for senior coaches to not only get ‘results’, but to do so in a way that reflects a club vision. This further demonstrates the complexity of coaching at the high-performance level. Our findings suggest that the NFC and subsequent content delivered on the coaching courses needs to better represent the requirements, expectations and perhaps even echo the pressures associated with the senior coaching role.

### Theme 3: Practice design

A fundamental priority for coaches in performance environments is the successful design and integration of performance preparation frameworks capable of supporting athletes in regulating behaviour in training and competition [61]. Accordingly, this final component of the interview presented an opportunity for participants to explore the practice strategies used, and why, in helping support the development of football skills.

#### Sub-theme 3.1: Approaches to practice design

When it came to identifying what type of practice strategies participants preferred, all favoured the need for ‘realistic’ or ‘representative’ environments. Here, we refer to such practice through the lens of ‘representative learning design’ [62]; defined as training activities that are representative of the informational constraints of competition [61,63]. It is thought that players who are exposed to representative learning designs will learn to ‘pick up’ key sources of information used to directly regulate their behaviour in competition environment [61]. This was a sentiment articulated by HC10 and HC7, respectively:

*“I think representative design is crucial to be able to get the learnings across efficiently and effectively*. *If training is not representative of the actual scenario faced on the weekend or the problems faced on the weekend, I think you’re going to miss the mark, to be honest.”*

*“Representative design is massive*. *Training has to look like the game. That doesn’t mean we play 11 v 11 in every session, but it means that generally the game is directional, there’s always opposition and more often than not, the attention is focused centrally towards a goal scoring point. 95% of our sessions will involve those principles. On a simplistic and fundamental level, training is designed so players are playing football competitively, that reflects the game.”*

These responses are consistent with FA’s position on practice design, who state that for ‘proper’ learning to occur, training exercises require ‘game-specific resistances’ (i.e., opponents) that replicate the actual football situation in which the problem occurred [10]. Despite this stance, FA do state that in certain circumstances when coaches have exhausted all other options for developing a player, isolated and unopposed training may be used [10]. Such a sentiment seemed to be shared by participants, who despite advocating for representative learning design, accepted that a place for unopposed practice remained:

*“Unopposed game patterns, which is something I use in the preseason working on what I call the ‘what if’ scenarios*. *I spend a lot of time in pre-season working on that, building a cohesion and I guess synergy*. *In the first instance, unopposed, maybe mannequins, but starting from different parts of the field and working through scenarios and options, just presenting them with alternatives. If this happens, there’s an alternative. And by repeating and repeating and repeating and repeating, firstly, without competition just patterns, they start building their own decision making based on the ‘what if’s’. Then, as you introduce a passive opponent and that passive opponent hypothetically leans right, then we go to option three, they go left, we play through the line beyond the press, and we do this.” [HC15]*

This perhaps highlights a theoretical inconsistency. For example, research suggests that practice designs containing specifying information leads to effective and efficient skill transfer from training to competition, whereas practice designs containing non-specifying information may lead to a more general transfer [64]. The majority of participants shared the belief that isolated and unopposed training had merit and would likely transfer into competitive games, a belief likely found on the assumption that breaking up tasks into isolated components is beneficial to enhance performance [65,66]. Although despite this belief, it was of note that most participants were well aware of the limitations of unopposed practice with regards to the development of football skill. Moreover, some participants mentioned that unopposed practice was implemented to cater to areas such as introducing new tactical concepts, manage player physical load, and increase confidence of players prior to a competitive match. This was exemplified by HC7:

*“I’m certainly not anti-unopposed practice, there’s just a place for them. If we play on a Sunday and we have one session between then and a Wednesday game, may be the best way to get a tactical idea out without compromising the loading [of players] is to do an 11 v 0 and an unopposed type of session. So, I will use them if that’s the best tool to get something out in a short period of time. But I think you neglect long term learning by doing anything unopposed*. *For me, a pre-set pattern is fragile. If I say, right, this is how we will build up to create a chance and this is the pattern that we work on repetitively unopposed at training. What happens when that doesn’t become available in the game? But if we train over and over again with the right level of opposition and problems to solve, we will create long term learning and problem solvers and collective problem solving cannot happen without putting them in problems to solve over and over and over again.”*

Such sentiment indicates that additional factors, like training and playing schedule, are likely to influence practice design approaches for senior football coaches. Although participants favoured representative design, the nature and complexity of the high-performance environment meant ideal training methodologies were compromised. Indeed, there are other ways of reducing the complexity of practice while preserving its representativeness (see [67]), and given that most participants recognised the limitations of unopposed practice for sustained learning, FA could consider integrating such ideas into the NFC, like manipulating constraints in practice to preserve functionality in representative ways. Practice, in other words, could be reduced in complexity through *simplification*, rather than *decomposition* [67], thereby offering greater theoretical coherence in senior coaches undertaking their accreditation processes.

#### Sub-theme 3.2: Theoretical sentiments associated with practice design

In this part of the interview, participants were asked if they were guided by a particular theoretical framework of practice design and player development. According to FA’s coaching philosophy, which is underpinned by an information-processing view of motor learning, ‘perception’, ‘decision’, and ‘execution’ are considered as three distinguishable, yet interconnected processes a player undertakes before completing a football action [7]. This appears to have influenced some participants, as noted in responses by HC8 and HC12, respectively:

***“**There is a literal chronological process of how decision making takes place in the brain, which is first you receive communication, so you perceive words and body language, it goes into your brain*. *Then your game insight, in other words, your knowledge, your experience as a player determines what solutions pop up in your brain and that you see as options. And then you obviously pick one and then you execute.”*

*The ‘perception’, ‘decision’, ‘execution’ is the single biggest failure of us coaches over my period [in Australian football]….we have to allow the players to make decisions, I don’t care what decision you make, as long as you make one*. *Execution is purely about repetition, executing something is the output of the first two [perception and decision making].”*

These responses suggest that at a superficial level, the information-processing view of motor learning presented by FA has influenced how some participants conceptualise player behaviour. By FA’s own account, the complexity of football requires players to rapidly select a particular response from a wide range of possible options and execute them under pressure [10]. Although this perspective on motor learning and skill acquisition still holds a strong position in player development models, ecological dynamics has emerged as a theoretical alternative in the explanation of learning and behaviour (for a detailed overview of its key tenets, see [68]). Despite the opposing theoretical stance of FA, it was of note that a handful of participants mentioned that an ecological dynamics rationale influenced their view of player development, practice design and their role in performance preparation. For example, when asked if they followed a particular theoretical framework, HC7 responded:

*“An ecological dynamics approach. You went through the [education] process without really being exposed to the theory around ecological dynamics and the constraints-led approach*. *[The NFC] didn’t feel right, and that coaching in the manner we were asked to do, with a lot of unopposed stuff, a lot of over-structured coaching felt really unnatural to me.”*

Despite miss-guided and uninformed criticisms, ecological dynamics offers a *blended* theoretical framework that brings together key ideas from ecological psychology and dynamic systems theory (see [68,69]). It is far from a ‘one-size fits all’ approach, offering sports practitioners with a theoretical positioning that explains behavioural emergence without relying on abstract mentalistic and mechanistic concepts. This helps guide the design of informationally rich and representative practice tasks (see [70]). Interestingly, these sentiments were noted by HC3:

*“There’s never going to be a secret of how you score, because it’s an emerging concept that depends not just on what you do, but on the opposition. So, for me, all these ideas of trying to come up with some mechanistic approach… misses the point*. *I see the game as a complex, dynamic system with the scoring of the goal an emergent concept that really depends on the interactions of many parts. I don’t control an individual and I don’t control the individual process, I don’t control it in microscopic detail. But what I need to do is ensure that there are certain forms of interactions which happen within that system that generate certain emergent behaviours. If a player or playing group can come up with an idea that maybe I was missing right then, if they come up with it themselves, it will stick. If I say it, it may or may not stick, because the process of conceiving something I think ingrains it in the mind much more so than passively receiving that information.”*

These findings, in conjunction with earlier sentiments regarding the rigidity of course content, demonstrate divergent theoretical perspectives on motor learning and control within the participants. Regardless of theoretical positioning, it is clear that FA need to consider supporting coaches in the exploration of theoretical concepts that resonate with them. This could take shape in the delivery of alternative theoretical frameworks and their implications for a coach’s role and practice design. Such diversity in the content of the NFC may thus foster ongoing exploration, not replication–a theme noted earlier as being diminished through the current delivery of rigid content.

## Conclusion

The aim of this study was to explore Australian football coaches’ perspectives of formal coach education delivered by FA. Doing so allowed us to shed light on how coaches conceptualise their role, and what practice strategies they employ to support player development. With regards to formal coach education, the findings indicated that while courses supported a coaches’ basic methodological and procedural practice (i.e., structuring and planning training), the Advanced Coaching pathway was largely ineffective in preparing coaches for the rigours of senior football. This was primarily due to the nature, structure, and delivery of the content, which led to sentiments of indoctrination and conformity. When discussing their role in high-performance settings, participants emphasised its complexity, requiring competence across multiple domains. This appeared to be under-appreciated in the content delivered as part of the accreditation process. Finally, when discussing what practice strategies participants used, most expressed a strong preference for designing practices that are ‘representative’ of the competitive environment. Though, some did mention that utility of unopposed practice in high-performance settings, albeit for reasons other than skill development.

Our overarching aim was to gain a deeper understanding of the coach education landscape in Australian football, helping foster the voice of Australian senior football coaches in discussing the much-debated topic of coach development and education. The findings demonstrate that FA faces challenges far beyond simple cosmetic upgrades to the structuring and delivering of content on their courses. They rather point to broader, systemic issues relating to the conceptual, theoretical, and practical foundations of the NFC and subsequent courses. If the intention of FA is to develop ‘world class’ coach development environments capable of effectively developing the behaviours, knowledge, and practices of Australian football coaches, then greater emphasis on delivering coach education in a manner that meets the needs of the coaching community is required. We hope that these findings encourage FA and researchers to continue engaging with the Australian coaching community and collectively seek ways to develop future iterations of the NFC and coach education programs, thus better supporting and enhancing the theoretical, philosophical, and practical orientations of Australian football coaches.
